# Systemic reaction to and interference with urine protein measurements after intravenous fluorescein injection

**DOI:** 10.1186/s12886-019-1276-x

**Published:** 2019-12-19

**Authors:** Annemieke Clementine Bouman, Damaris de Kruijf, Judith Maria Antonia Emmen, Thijs Clemens David Rettig, Maria Jolande Charlotte Kooijman-de Groot, Miriam Christina Faes

**Affiliations:** Department of geriatrics, Amphia hospital, Langendijk 75, 4819 EV Breda, the Netherlands

**Keywords:** Fluorescein angiography, Anaphylactic reaction, Interference, Laboratory analyses, Urinalysis

## Abstract

**Background:**

Fluorescein angiography is an important and frequently used diagnostic tool in ophthalmological practice. In this case report we describe a patient who experienced an anaphylactic reaction after the injection of fluorescein. Furthermore, we report an interference with laboratory testing by fluorescein in this patient and summarize the literature on this topic.

**Case presentation:**

An 86-year old Caucasian woman undergoing fluorescein angiography due to suspected peripapillary neovascularizations collapsed after the injection of fluorescein. The patient developed an anaphylactic reaction. With fluid resuscitation and oxygen therapy, the patient regained consciousness after a few minutes. The patient was admitted to the geriatric ward for observation, and routine blood and urine tests were performed. Urine protein concentration appeared to be falsely increased as a consequence of disturbance of the laboratory analysis by the presence of fluorescein.

**Conclusions:**

Serious complications can occur with fluorescein angiography, such as an anaphylactic reaction. In the case of anaphylaxis appropriate supportive measures including the use of oxygen and epinephrine (e.g. EpiPen), should be available to prevent morbidity and mortality from this test. Furthermore, these potential complications should be taken into consideration when choosing the healthcare setting for fluorescein angiography, such as the immediate availability of an acute medical team.

Several studies have demonstrated the interference of laboratory analyses by fluorescein. The majority of these studies were published 10 to 30 years ago. By presenting this case, the authors hope to bring renewed attention to this phenomenon among clinicians, as falsely increased or decreased laboratory values can result in unnecessary diagnostics and/or therapy.

## Background

Fluorescein angiography is an important diagnostic tool in ophthalmological practice. This tool was first described by Novotny and Alvis in 1961 [1]. Intravenously injected fluorescein exposes the retinal and choroidal vasculature. The most important indications for fluorescein angiography are diabetic retinopathy, suspected neovascularizations in macular degeneration, and retinal vascular occlusions [2]. Fluorescein angiography is executed approximately 1000 times a year in an average ophthalmological outpatient clinic in the Netherlands.

Fluorescein angiography is not without risk. The most common harmless side effect is nausea in 1–3% of patients; in 0.4–0.6%, this is accompanied by vomiting. However, more serious complications, such as myocardial infarction, asystole, anaphylaxis, and death have also been reported [3]. The incidence of anaphylactic reactions after fluorescein angiography differs between studies; incidences between 0.008 and 0.05% have been described [4].

In this case report, we describe a patient with an acute reaction to fluorescein. Furthermore, we describe the potential influence of fluorescein on laboratory test results.

### Case description

Patient A, an 86-year-old woman, visited the ophthalmological outpatient clinic for fluorescein angiography, due to suspected peripapillary neovascularization. Ophthalmic examination showed a suspected lesion and a hemorrhage near the optic disc (subretinal), and the SD-OCT (spectral domain optical coherence tomography) of the macula showed a macula pucker.

She had a medical history of rheumatoid arthritis, bilateral hip replacement, various operations for variceal veins, and surgical correction of an uterine prolapse. The patients’ history did not report asthma or renal function disturbances. She used calcium carbonate/cholecalciferol, folic acid, methotrexate, and timolol and hypromellose eye drops. She was allergic to sulfasalazine and acetylcysteine.

Immediately after intravenous injection of 5 ml of 10% sodium fluorescein, the patient became sick and lost consciousness. The rapid response team arrived on site and found an unresponsive, pale, and sweating patient with reduced consciousness (E3M6V5). Her airway was free, and the respiration rate was 20 times per minute. Peripheral oxygen saturation was 83%, and on auscultation of the lungs, normal vesicular breath sounds were heard. Blood pressure was 81/58 mmHg and the heart rate was irregular with a frequency of 150 beats per minute. ECG showed atrial fibrillation. The temperature was 36 °C, and the glucose concentration was 7.7 mmol/l. The patients’ skin did not show any signs of redness or urticaria.

The laboratory results demonstrated metabolic acidosis (pH 7.30) caused by an increased level of lactic acid of 4.7 mmol/l. This was explained by reduced tissue perfusion as a consequence of hypotension.

After the administration of oxygen via a nonrebreathing mask, the oxygen saturation was restored to 97%. The patient was given 500 cc of 0.9% sodium chloride solution intravenously. Within a few minutes, she regained consciousness, and after 20 min, she was able to communicate adequately again. Blood pressure normalized, and atrial fibrillation spontaneously converted to sinus rhythm. Epinephrine was not administered, as the anaphylactic reaction improved rapidly with oxygen and intravenous fluids. Five hours after the event, the blood lactic acid level and pH were normalized.

The patient was admitted to the geriatric ward for observation, and routine blood and urine tests were performed. The total urine protein concentration appeared to be considerably increased (Table 1, urine sample 1). The urine had a distinct yellow-green color.

**Table 1:** Results of the two urine samples

	Reference values	Urine sample 1	Urine sample 2
Urine, total protein	g/L	1.95	0.047
Urine, creatinine	mmol/L	2.7	2.8
Urine, protein/creatinine ratio	< 0.15 g/10 mmol	7.22	0.17
Urine, albumin	0–20 mg/L	311.4 (= 0.3 g/L)	4.7
Urine, albumin/creatinine ratio	0.0–3.5 mg/mmol	115.3	1.8

The patient recovered, and the next day, she was discharged in a sound condition.

Urinalysis was repeated 6 days after the administration of fluorescein. The previously found abnormalities had almost disappeared (Table 1, urine sample 2).

## Discussion and conclusions

The patient most likely suffered from an anaphylactic reaction to fluorescein [5]. Fluorescein angiography is a relatively safe diagnostic tool. However, in a minority of cases, more serious side effects can occur [3, 6]. Cases have been described of patients with hypotension and syncope after the administration of fluorescein. These were considered anaphylactic reactions. The characteristic rash was only observed in a subset of patients with an anaphylactic reaction. Our patient did not have a rash, but she did experience hypotension, syncope, nausea, and vomiting. Therefore, the most likely diagnosis is an anaphylactic reaction to fluorescein. Atrial fibrillation was considered a reaction to the hypotension and it did not reoccur.

In this case treatment with oxygen and intravenous fluids was sufficient. However, in case of an anaphylactic reaction, intravenous or intramuscular epinephrine is the treatment of choice. In the United States, EpiPen is mandatory in clinics where fluorescein angiography is performed. High-flow oxygen should be administered to patients experiencing respiratory symptoms or hypoxemia. Patients who are hypotensive should have fluid resuscitation. After the treatment of an anaphylactic reaction, an observation period of 4–6 h should be considered for all patients because the reaction might recur as the effect of epinephrine wears off and because of the risk of a biphasic reaction (incidence 1–20%) [5].

A discrepancy between the results of the total protein and the albumin concentration in the urine was found. None of the patients’ prescribed medication could cause this discrepancy. Based on the high total urine protein concentration of 1.95 g/L, a much higher albumin concentration than 0.3 g/L was expected because albumin is the most prevalent protein in urine. The presence of fluorescein in the urine appeared to disturb the total urine protein analysis, which resulted in an incorrectly increased result. Urinary protein was measured using a turbidimetric method in which benzethonium chloride precipitates urinary protein in an alkaline medium. Fluorescein did not interfere with the analysis of (micro-)albumin in urine and could therefore be used as an alternative method.

The interference of fluorescein with laboratory analyses is method dependent and is described for analyses that use fluorescein labels or fluorescein detection or for analyses that measure close to the excitation or emission frequency of fluorescein [7]. Several studies have described the interference of fluorescein with laboratory analyses. However, the majority of these studies are approximately 10 to 30 years old. Therefore, it is important to bring renewed attention to this phenomenon, as falsely increased or decreased laboratory values can result in unnecessary diagnostics and/or therapy. In 1991, Koumantakis et al. reported interference of fluorescein with urinary protein measurements. In nine patients, urinary samples were collected before and after the injection of fluorescein. Fluorescein was found to increase urinary protein. Urinary protein was measured using the benzethonium chloride method. Additionally, fluorescein also interfered with the measurement of creatinine in the urine. The authors of this study advise delaying urinary testing at least 24 h after the injection of fluorescein [8].

In 1989, Bloom et al. performed a study in which the effect of fluorescein on several serum and urine chemistry tests was investigated. In four healthy volunteers, a panel of blood and urine tests was performed before and at several time points after the intravenous injection of 5 mL of fluorescein sodium. Serum creatinine and protein levels were disturbed in the sample taken 5 min after injection. Interference of fluorescein with levels of cortisol, digoxin, and thyroxin was established up to 12 h after the injection of fluorescein [9]. A difference in blood glucose measurements with at home glucose meters after fluorescein angiography has also been described. Although the difference reached statistical significance, it did not appear to be clinically significant, as the difference did not result in changes in insulin dosage [10]. In another study, falsely increased levels of creatinine were found 20 min after the injection of fluorescein. The interference was dependent on the type of method of creatinine measurement used [11]. Flattem et al. described a case in which the measurement of homocysteine in the blood, drawn 1 hour after fluorescein angiography, was disturbed by the presence of fluorescein [12]. In a study by Renoe et al., blood samples were spiked with fluorescein. In these blood samples, the measurements of magnesium and total protein levels in serum were influenced by fluorescein [13]. McClellan et al. reported the interference of fluorescein with several laboratory analyses. Magnesium, phosphorus, and total protein were artificially decreased. The creatinine kinase MB fraction was increased [14].

As described above, in the past several studies showed the interference of various laboratory tests with the use of fluorescein. Although only a few laboratory tests are affected by the presence of fluorescein, and although tests continuously undergo improvements to minimize possible inference, the potential interference of fluorescein should be considered. Fluorescein is cleared from the blood mainly by the kidneys. Therefore, in patients with renal function impairments, which is the case in many of the patients with diabetes or hypertension undergoing fluorescein angiography, the interference might persist longer than in healthy individuals. In these patients, laboratory analyses should be postponed even more than 24 h [9, 14].

In conclusion, potentially serious complications can occur with fluorescein angiography, such as an anaphylactic reaction. Patients should be properly informed in order to understand the risk of fluorescein angiography, the clinical need for this test and the potential treatments that are available in the clinic in the case of adverse reactions. It is important to take potentially serious complications into account when choosing the setting for fluorescein angiographies. An acute medical team must be quickly available on site.

Fluorescein can interfere with laboratory tests. Clinicians should be aware of the phenomenon and keep this in mind when planning laboratory analyses in the days after fluorescein angiography. Laboratory analyses should be postponed by at least 24 h after fluorescein angiography and longer in cases of renal function impairment. If laboratory analyses are needed shortly after the injection of fluorescein, it is important to check with the laboratory specialist in terms of which analyses might be disturbed as well as the possible alternatives.

## Data Availability

Not applicable.

## Abbreviations

SD-OCT: Spectral domain optical coherence tomography
